# Quantitative powder diffraction using a (2 + 3) surface diffractometer and an area detector

**DOI:** 10.1107/S1600576721006245

**Published:** 2021-07-16

**Authors:** Giuseppe Abbondanza, Alfred Larsson, Francesco Carlá, Edvin Lundgren, Gary S. Harlow

**Affiliations:** a Division of Synchrotron Radiation Research, Lund University, 221 00 Lund, Sweden; b NanoLund, Lund University, 211 00 Lund, Sweden; c Diamond Light Source, Didcot OX11 0DE, United Kingdom; d Department of Chemistry, University of Copenhagen, 2100 Copenhagen, Denmark

**Keywords:** powder diffraction, Rietveld refinement, angle calculations, grazing incidence, area detectors

## Abstract

X-ray diffractometers primarily designed for surface X-ray diffraction are often used to measure the diffraction from powders, textured materials and fiber-texture samples in 2θ scans. Unlike in high-energy powder diffraction, only a fraction of the powder rings is typically measured, and the data consist of many detector images across the 2θ range. Such diffractometers typically scan in directions not possible on a conventional laboratory diffractometer, which gives enhanced control of the scattering vector relative to the sample orientation. There are, however, very few examples where the measured intensity is directly used, such as for profile/Rietveld refinement, as is common with other powder diffraction data. Although the underlying physics is known, converting the data is time consuming and the appropriate corrections are dispersed across several publications, often not with powder diffraction in mind. This paper presents the angle calculations and correction factors required to calculate meaningful intensities for 2θ scans with a (2 + 3)-type diffractometer and an area detector. Some of the limitations with respect to texture, refraction and instrumental resolution are also discussed, as is the kind of information that one can hope to obtain.

## Abstract 2

Conversion of 2D detector images measured on a (2 + 3) surface diffractometer to meaningful intensities that can be used for quantitative analysis of powdered and textured materials is discussed.

## 1. Introduction   

Since the early studies by Debye & Scherrer (1916 ▸), powder X-ray diffraction (PXRD) has become a well established characterization technique. It has proved to be a fundamental tool for phase identification and structure determination of materials. Quantitative analyses of PXRD data enable access to information such as size, strain and stress of the crystallites, the number of different phases in multi-phase materials, and atomic and unit-cell parameters. PXRD data quality improves significantly when synchrotron X-ray beams are employed, which provide a high photon flux, enhanced collimation, tunable energies and a superior angular resolution. In surface X-ray diffraction (SXRD), an experimental setup composed of a (2 + 3) diffractometer and an area detector is used at some beamlines. The (2 + 3) diffractometer was presented by Vlieg (1997 ▸) and its combination with an area detector was explored by Schlepütz *et al.* (2005 ▸). Although this setup was originally designed for single-crystal SXRD, it can also be used in PXRD and in grazing-incidence X-ray diffraction (GIXRD) by rotating the detector about the diffractometer center in longitudinal and equatorial 2θ scans, across the Debye–Scherrer cones. In this scenario, the major benefit of the (2 + 3) diffractometer is control over the orientation of the sample and of the detector (and thus of the scattering vector), which enables convenient investigations of specimen textures and preferred orientations. This experimental setup is thus well suited for a wide range of sample types with an extended face, such as thin polycrystalline films on substrates. The control over the grazing-incidence angle and thus over the X-ray penetration depth enables the study of layered materials and buried interfaces. Furthermore, the relatively high resolution of the instrument (compared with bench-top diffractometers) enhances the study and the identification of multi-phase systems.

The phenomena involved in PXRD are well known and have been extensively studied in the past century. A significant step forward in the analysis of PXRD data was the Rietveld method (Rietveld, 1966 ▸, 1967 ▸; Young, 1993 ▸; van Laar & Schenk, 2018 ▸). Such refinement is usually performed on PXRD patterns where the intensity is plotted as a function of 2θ. Therefore, with the (2 + 3) setup at the I07 beamline (Nicklin *et al.*, 2016 ▸), it is necessary to integrate the two-dimensional data collected by the area detector into a one-dimensional pattern. Furthermore, a series of intensity corrections should be applied to the measured intensities, to obtain the structure factors that depend on the underlying crystallography of the sample.

Area detectors with fixed position have been used extensively in powder diffraction, *e.g.* in the mapping of grain boundaries (Poulsen *et al.*, 2001 ▸) and in texture analysis (Wenk & Vasin, 2017 ▸). Furthermore, software meant for the analysis of two-dimensional diffraction data has been developed. For instance, *Fit2D* (Hammersley, 2016 ▸) and *pyFAI* (Kieffer & Wright, 2013 ▸) enable the integration of two-dimensional data to a one-dimensional pattern only for detectors with fixed position. The software *BINoculars* (Roobol *et al.*, 2015 ▸; Drnec *et al.*, 2014 ▸) is tailored toward the analysis of surface X-ray diffraction data and is used to assign the intensity detected by every single pixel of an area detector to a bin in the three-dimensional reciprocal space. This means that it is possible to reduce the powder diffraction data collected by a (2 + 3) diffractometer with this software, although it is computationally expensive and requires a backend.

**Figure 1:**
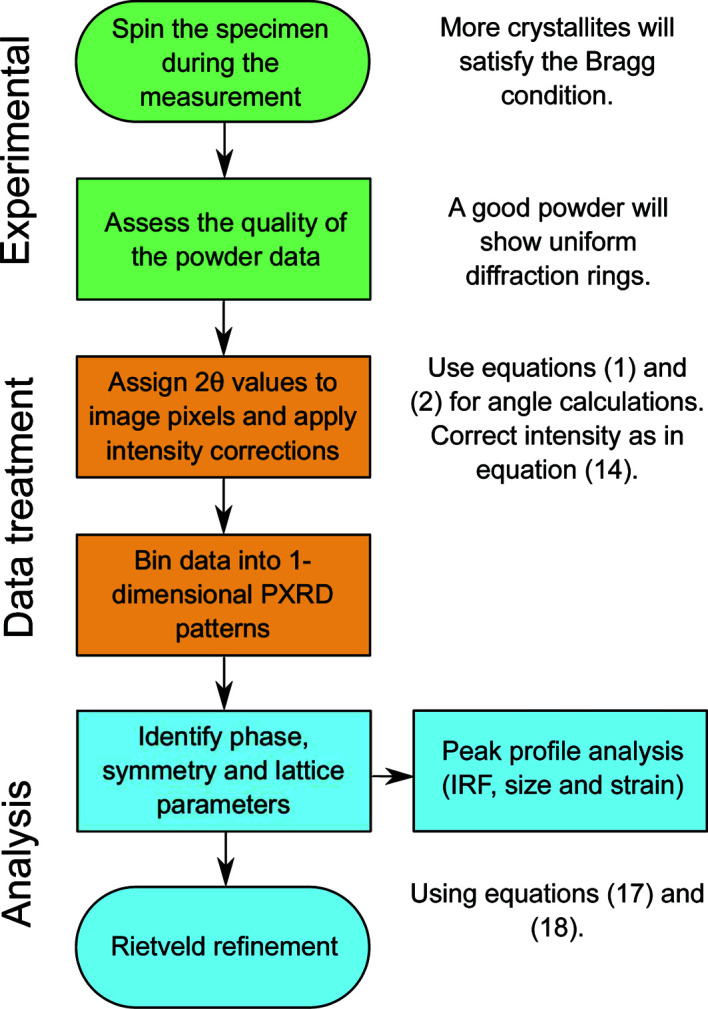
Flowchart outlining the steps and the relevant equations involved in the investigation of powdered samples from the experiment to the determination of the structure.

In this work, we present the calculations and the correction factors needed to extract quantitative information from 2θ scans with (2 + 3) diffractometers and area detectors. The calculations are part of a process intended for the characterization of polycrystalline materials, as illustrated by the flowchart in Fig. 1 ▸. We assess the validity of the calculations and of the correction factors by refining an LaB_6_ reference sample. Furthermore, we calculate the instrumental resolution function of the setup and compare the integrated intensities collected in different experimental geometries, namely capillary in transmission, grazing incidence and Bragg–Brentano.

## 2. Experimental setup   

The experimental work was conducted at beamline I07, a hard X-ray (8–30 keV) high-resolution diffraction beamline at Diamond Light Source, UK (Nicklin *et al.*, 2016 ▸). The X-ray beam had an energy of 20 keV and a size of 100 µm vertically and 200 µm horizontally. To record the powder diffraction intensities, a Huber (2 + 3) diffractometer (Vlieg, 1998 ▸) and a Pilatus 100K detector were used.

**Figure 2:**
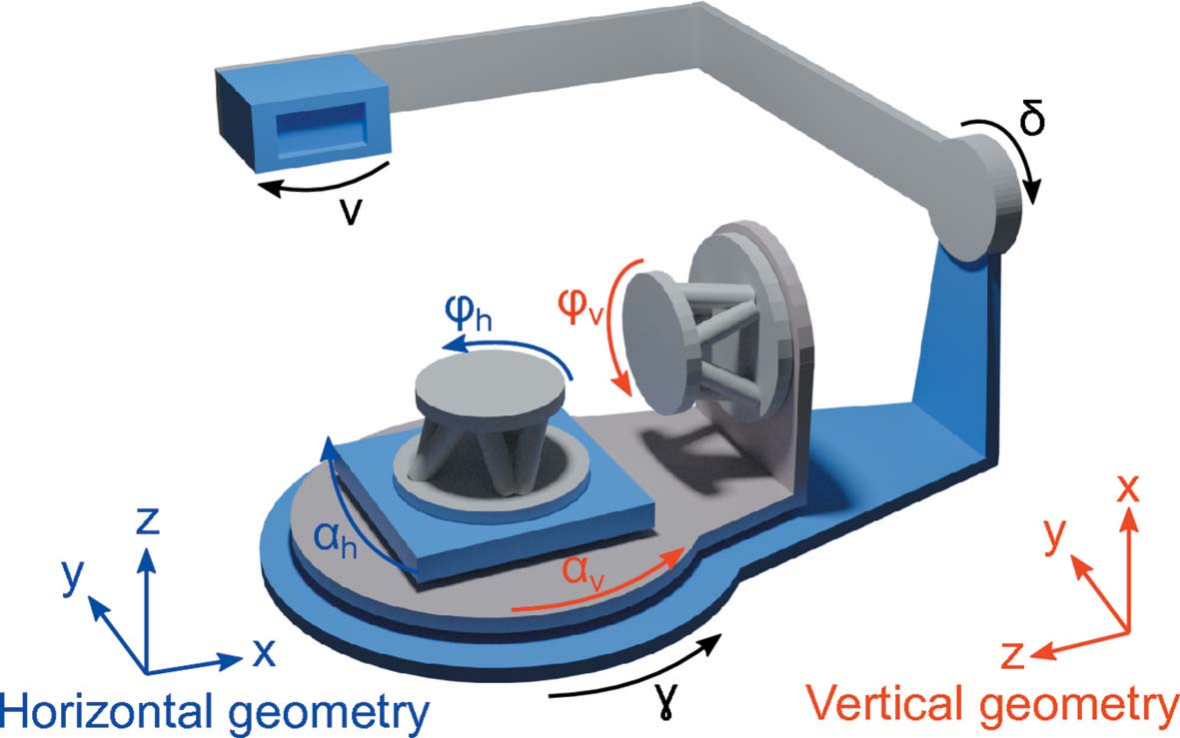
Schematic of a (2 + 3) diffractometer. The arrows point towards the positive direction of rotation in the case of horizontal (blue) or vertical (red) geometry and for the detector (black).

A schematic diagram of the Huber (2 + 3) diffractometer is shown in Fig. 2 ▸. The coordinate frame of reference depends on whether the sample geometry is horizontal (blue) or vertical (red). In both modes of operation the sample is mounted on a hexapod (Micos), which allows scanning of the sample translations and rotations for the initial alignment. The grazing-incidence angle of the synchrotron beam onto the sample surface and the azimuth are given by the rotation of α_h_ and φ_h_ in the horizontal geometry, and by α_v_ and φ_v_ in the vertical geometry.

The three detector circles are fully independent of the two sample circles and allow for radial scans in the horizontal plane (γ) or out of the horizontal plane (δ), and rotation of the detector around its surface normal (ν).

The Pilatus 100K area detector consists of an array module of 487 × 195 pixels with size 172 × 172 µm, resulting in an active area of 83.8 × 33.5 mm, and it can detect photons in an energy range of 3–30 keV with a dynamic range of 2^20^ (Kraft *et al.*, 2009 ▸). A set of slits is positioned between the detector and the sample. The primary function of the slits is to reduce the background and isolate the signal originating from the sample at the center of rotation of the instrument. This is particularly useful in surface diffraction/scattering experiments where the sample is contained in a sample environment with entrance and exit windows that are producing high-intensity background. The sample-to-detector distance and the slit positions are adjustable and can be adapted according to the setup and the experimental parameters to eliminate window-generated background (see Appendix *A*2) and optimize the angular resolution.

Owing to the size of the detector and to the energy range available in this experimental setup, the detection of whole Debye–Scherrer rings in an image is not feasible. Therefore, the detector arm is usually scanned across the diffraction cones and small fractions of the Debye–Scherrer rings are detected.

## 3. Angle calculations   

**Figure 3:**
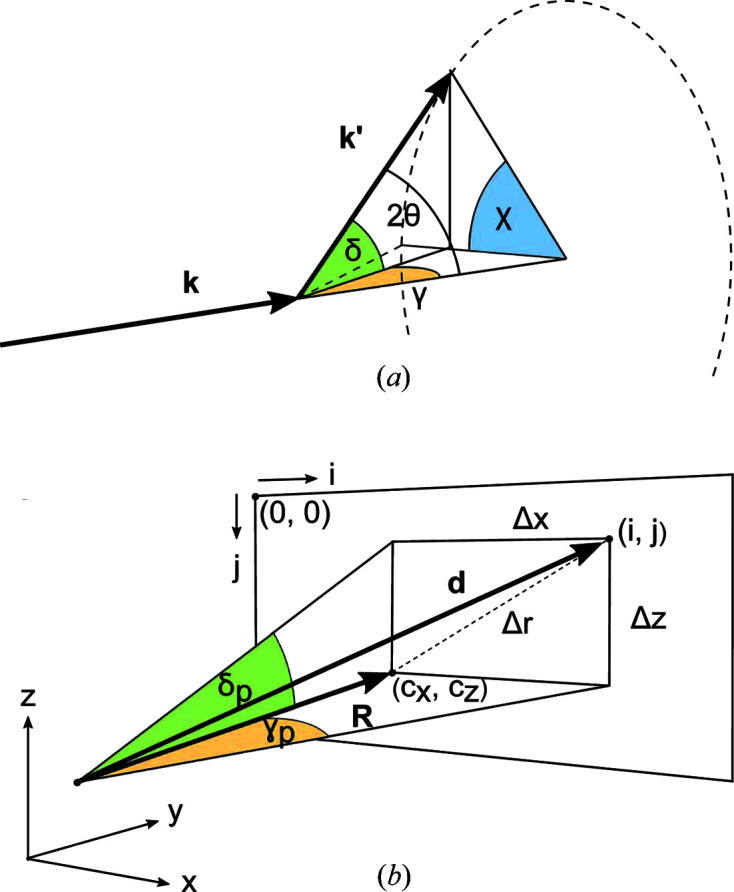
(*a*) Schematic representation of the angles γ, δ, 2θ and χ, subtended by the incoming beam wavevector **k** and a generic scattering wavevector **k**′ and (*b*) diagram showing the offsets Δ*x* and Δ*z* from the detector center of a generic (*i*, *j*) pixel. The vector **d** (where |**d**| is the distance of the pixel from the diffractometer center) subtends the angles δ_p_ and γ_p_.

With the setup described above, the Debye–Scherrer rings are detected by scanning the detector arm along γ and δ. The sequence of detector images collected during a scan can be integrated and processed to obtain diffracted intensities as a function of the diffraction angle 2θ and the azimuthal angle χ, illustrated in Fig. 3 ▸(*a*). A detailed derivation of these angles for every pixel of an area detector is given in Appendix *A* [App appa]. Accordingly, 2θ and χ are expressed as 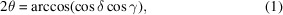


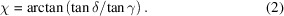



**Figure 4:**
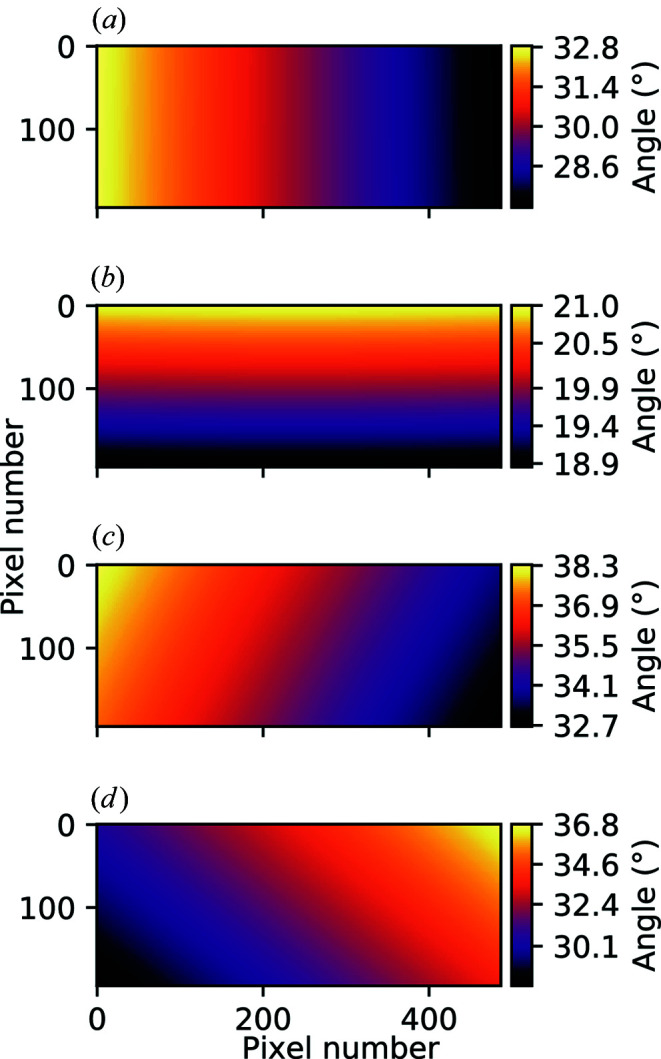
2D plots of the angle values assigned to each pixel of a Pilatus 100K detector positioned at γ = 30°, δ = 20° and *R* = 897 mm. The plots show γ_p_ (*a*), δ_p_ (*b*), 2θ (*c*) and azimuthal χ_p_ (*d*).

Fig. 3 ▸(*b*) shows the angles γ_p_ and δ_p_ subtended by a generic detector pixel (*i*, *j*), located at a certain distance *d* from the diffractometer center. Once the coordinates (*x*_p_, *y*_p_, *z*_p_) of this pixel are determined using the rotation matrices shown in Appendix *A* [App appa], it is possible to calculate γ_p_ and δ_p_ as follows: 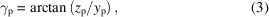


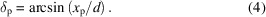
Therefore, equations (3) and (4) can be substituted into equations (1) and (2) to produce angle maps like those shown in Fig. 4 ▸. The maps in Fig. 4 ▸ are calculated for the case where γ = 30° and δ = 20°, assuming a detector distance of 897 mm, which is the distance at which the data were collected in this work.

## 4. Intensity correction factors   

The intensity of a powder diffraction pattern can be expressed as 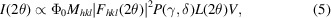
where Φ_0_ is the incident photon flux, *M*_*hkl*_ is the multiplicity (*i.e.* the number of symmetry-equivalent reflections contributing to a single peak), *F*_*hkl*_ is the structure factor, *P*(γ, δ) is the polarization factor, *L*(2θ) is the Lorentz factor and *V* is the sample volume from which the diffracted intensity arises. Deviations from this are often due to the presence of a preferred orientation or crystal texture.

### 4.1. Polarization factor   

**Figure 5:**
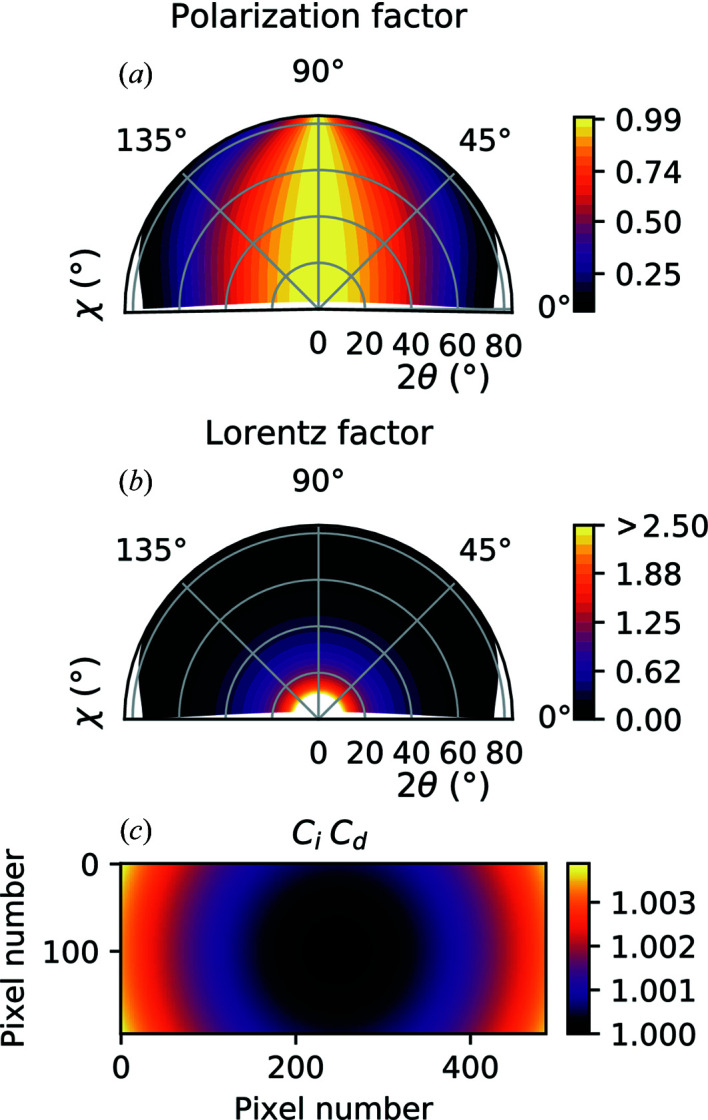
Intensity correction factors calculated at a distance of 897 mm from the diffractometer center. Polar contours of polarization factor (*a*) and Lorentz factor (*b*), as a function of the diffraction and the azimuthal angles. Flat-detector correction calculated for a Pilatus 100K with center pixel coordinates (*c*_*i*_, *c*_*j*_) = (246, 100) (*c*).

The X-ray beam produced by the I07 undulator has a strong horizontal polarization (Nicklin *et al.*, 2016 ▸) and therefore the scans in the horizontal plane (*i.e.* γ scans) are more affected by this factor than the scans out of the horizontal plane (*i.e.* δ scans). For this reason it is more convenient to express the polarization factor *P* as a function of (γ, δ) using the following expression which has been presented by several authors (Vlieg, 1997 ▸; Schlepütz *et al.*, 2005 ▸):
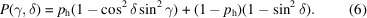
The polar plot in Fig. 5 ▸(*a*) illustrates the polarization factor as a function of 2θ and χ. This factor varies significantly in γ scans (*i.e.* when χ = 0°), while it is fairly constant for δ scans (*i.e.* when χ = 90°).

In high-energy X-ray diffraction, where the beam energy is usually in the 60–80 keV range and the whole Debye–Scherrer rings are detected, this factor is often neglected. With high energies, although the beam polarization is almost entirely horizontal (*e.g.* 98%), the angular-dependent decay does not have a significant impact. Assuming a detector distance of 1.5 m and that a large flat-panel detector is used (*e.g.* 40 × 40 cm), the highest **q** accessible has an absolute value of approximately 5.8 Å^−1^. If the beam energy is 80 keV, the corresponding 2θ angle will be 8.2°, and the highest drop in intensity due to the polarization on the detector horizon can be calculated using equation (6) and equals 4%. This small drop in intensity becomes even less significant when the intensity is integrated over the whole Debye–Scherrer ring. When lower energies are used (*e.g.* 20 keV), the 2θ angle corresponding to a scattering vector magnitude of 5.8 Å^−1^ is 33.2°. When the detector arm of a (2 + 3) diffractometer is scanned horizontally (*i.e.* δ = 0°) and reaches a γ value of 33.2°, the polarization has dropped by 31%. This decay must be taken into account, especially since only a fraction of the Debye–Scherrer rings are detected.

### 4.2. Lorentz factor   

A detailed derivation of the Lorentz factor was given by Buerger (1940 ▸), who defined it to be proportional to the time that a reflection stays in the Bragg condition. The Lorentz factor depends on the type of experiment performed and especially on the scanning variable employed to detect reciprocal space (Vlieg, 1997 ▸). For instance, in single-crystal diffraction, the integrated intensity is measured by rotating the sample over the entire width of a reflection while the detector position is fixed, in what is known as a rocking scan or Φ scan. In that case, the measured intensities need to be corrected by a geometrical factor, *i.e.* the Lorentz factor, that expresses the relative time spent by each point in reciprocal space in the reflecting position during the Φ scan. In PXRD, instead, the detector is rotated while the sample position is stationary, and thus there is virtually no access to reciprocal-space volume. However, owing to the random orientation of the crystallites, a powder can be seen as a single crystal that is rotated along the Φ axis and an axis orthogonal to the Φ rotation.

**Figure 6:**
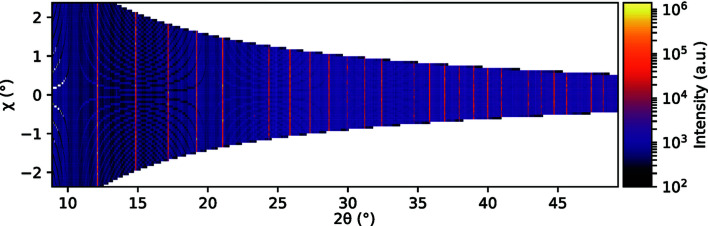
Diffracted intensity as a function of 2θ and χ originating from a NIST LaB_6_ SRM 660c sample encapsulated in a 1 mm-diameter borosilicate capillary. The data were collected in the Debye–Scherrer geometry by scanning the detector in the horizontal plane (γ scan).

In PXRD, the Lorentz factor *L* is the product of three terms. The first term is the ‘Darwinian’ single-crystal part (Darwin, 1922 ▸), which accounts for changes in integration volume as a function of 2θ: 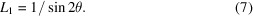



The second term is proportional to the fraction of the diffraction ring detected for different 2θ values. If we express the radius of the base of a generic Debye–Scherrer cone as 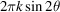, the fraction recorded by the detector is 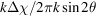 and therefore the second term will be proportional to 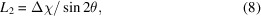
where Δχ is the range of azimuthal χ values accessible by the detector at a given γ and δ and can be considered constant (Als-Nielsen & McMorrow, 2011 ▸). This effect is evident in Fig. 6 ▸, where the diffraction intensity from a borosilicate capillary containing NIST LaB_6_ SRM 660c is plotted as a function of 2θ and χ. Here, the range of accessible χ values decreases significantly with the increase of 2θ.

The third term is proportional to the number of lattice points observable at the same time and therefore to the circumference of the base-circle of the Debye–Scherrer cones. If we denote a particular reciprocal lattice vector with **G**_*hkl*_, this circumference is 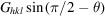 = 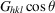. Under the assumption that the crystallites and thus their reciprocal-lattice points are homogeneously distributed on the Ewald sphere, this term is proportional to 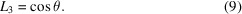
Note that *L*_3_ is not proportional to the possible permutations of (*h*, *k*, *l*) since these are already accounted for by the multiplicity factor *M*_*hkl*_ in equation (5). The Lorentz factor is given by the product *L*_1_ *L*_2_ *L*_3_ as in equation (10), which is rearranged in equation (11): 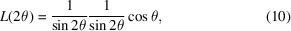


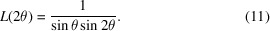



As shown in Fig. 5 ▸(*b*), the Lorentz factor has a rather quick decay as a function of 2θ, regardless of the azimuthal angle χ. It is perhaps the most significant intensity correction that needs to be applied to experimental data, especially at small 2θ.

### 4.3. Flat-detector corrections   

**Figure 7:**
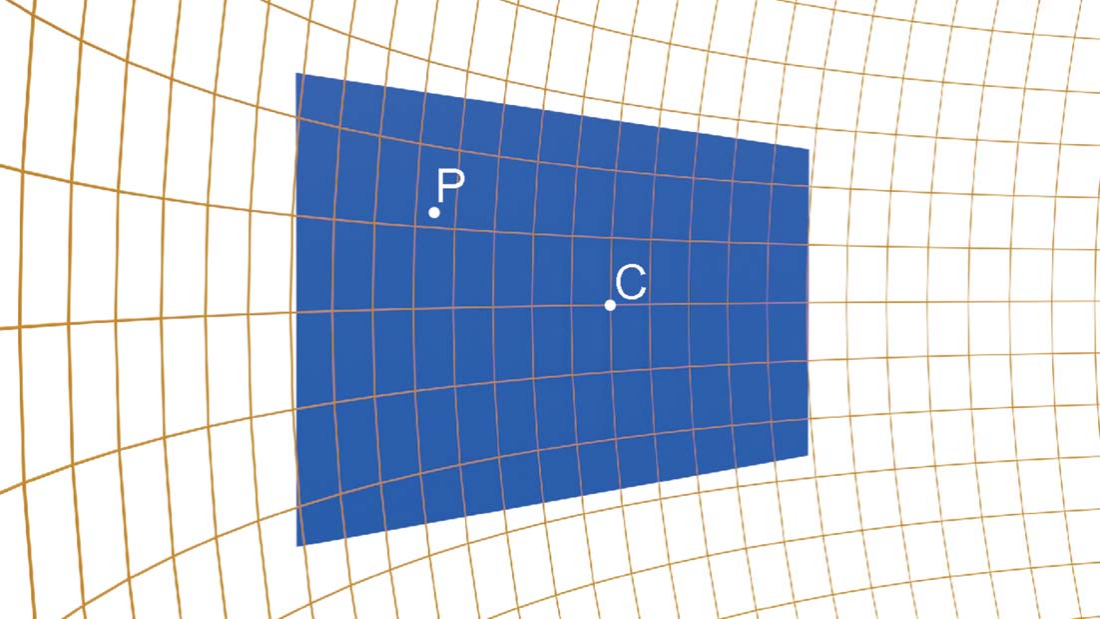
The flat surface of the Pilatus 100K (blue rectangle) intersects a sphere centered in the diffractometer (orange frame) at the point C (the detector center). A generic diffracted beam would impinge on the point C normally and subtend a non-normal angle with any other generic point P.

In addition to the polarization and the Lorentz factor, the decrease of the subtended solid angle for the different pixels has to be taken into account. Two contributions normally describe this change. The first is due to the fact that pixels away from the detector center are also further away from the diffractometer center, as depicted in Fig. 7 ▸. To account for this change in distance the measured intensities should be multiplied by 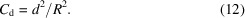

*C*_d_ is greater than unity for every pixel of the detector except the detector center C, where *d* = *R*. The second contribution is due to the non-normal incidence of the scattered beam owing to the flat detector surface and is given by Schlepütz *et al.* (2011 ▸):
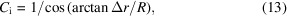
where Δ*r* = (Δ*x* ^2^ + Δ*z* ^2^)^1/2^.

For a better visualization of these correction factors, the product *C*_d_ *C*_i_ is plotted in Fig. 5 ▸(*c*) for the same detector distance *R*. The correction becomes more significant close to the edges of the detector image, where it exhibits a maximum change of about 0.35% from the detector center. The significance of this correction increases as the detector distance decreases or as the detector size increases.

### 4.4. Transmission corrections   

The sample volume that gives rise to diffracted intensity depends on the geometry of the experiment and is often a function of the scattering angle 2θ, the incidence angle α, and the angle between the sample surface and the diffracted beam γ = 2θ − α. The diffracting specimen volume is often modeled as an exponential aberration. Table 1 ▸ summarizes this correction for some common experimental geometries. As a general remark, the interaction volume is proportional to the area of illuminated sample, *A*, and is inversely proportional to the linear absorption coefficient, μ. Furthermore, note that refraction leads to a reduction of the penetration depth and thus the interaction volume when the incidence angle is below the critical value. This effect is well described by Feidenhans’l (1989 ▸), in a formalism that considers evanescent waves and their penetration into surfaces.

### 4.5. Summary of intensity corrections   

The corrected intensity is given by 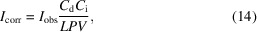
where *I*_obs_ is the observed intensity.

## 5. Peak profile analysis   

Analysis of the line-profile shape is useful for the determination of crystallite size and strain of a specimen. However, the width of a diffraction peak is a function of 2θ and it depends not only on specimen properties but also on experimental conditions and sample size. Furthermore, instrumental features such as monochromators, collimating and refocusing optics, slits, beam divergence and energy bandwidth influence the broadening of diffraction peaks (Gozzo *et al.*, 2006 ▸).

As the mentioned causes of broadening have either Gaussian or Lorentzian nature, the most used fitting functions for PXRD peaks are based on Voigt or pseudo-Voigt line shapes. The FWHM of the Voigt profile depends on the widths of the associated Gaussian and Lorentzian components Γ_G_ and Γ_L_ (Thompson, Cox & Hastings, 1987 ▸; Thompson, Reilly & Hastings, 1987 ▸).

### 5.1. Gaussian and Lorentzian broadening   

The Gaussian widths contain information on the instrumental resolution function (IRF) and on the sample strain. An analytical description of the IRF was given by Caglioti *et al.* (1958 ▸): 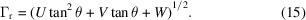
Specimen contributions to the Gaussian widths are the expression of crystal defects, dislocations and deformation of the unit cells, in what is known as inhomogeneous strain broadening: 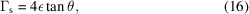
where the root mean square strain ε is a coefficient that depends on the elastic compliance and the mechanical properties of the specimen. Since this contribution is proportional to 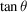, some authors have merged the strain contribution into equation (15). For example, Thompson, Cox & Hastings (1987 ▸) incorporated the coefficient ε into the constant *V* and Wu *et al.* (1998 ▸) into the constant *U*. In this work we adopt the solution presented by Thompson, Reilly & Hastings (1987 ▸), where the Gaussian width broadening is expressed as 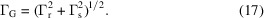
The Lorentzian widths Γ_L_ take into account the spectral bandwidth of the source and the sample crystallite size through the following equation (Cox, 1991 ▸): 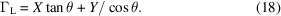
The *X* coefficient depends on the monochromating optics and it is of the order of magnitude of 10^−4^ for most of the synchrotron beamlines where Si(111) crystals are employed. The dependence of the bandwidth term on 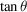 can be derived by differentiating Bragg’s law. The second term in equation (18) is the Scherrer crystallite size contribution. Here, *Y* = *K*λ/*D*, where *K* is a dimensionless shape factor (generally close to unity), λ is the wavelength of the X-ray radiation and *D* is the crystallite size. One should remember that the use of equation (18) relates to the size of the coherent diffraction domains rather than the size of the crystallites *per se* (Scherrer, 1912 ▸; Patterson, 1939 ▸; Hargreaves, 2016 ▸).

Equation (18) can be rearranged as 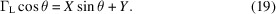
Plotting 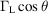 against 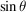 in equation (19) would produce a line where the intercept depends on the crystallite size while the slope depends on the beam spectral bandwidth. Such a plot is known as a Williamson–Hall plot (Williamson & Hall, 1953 ▸) and an example of such analysis is given in Section 6.1.

### 5.2. Beam footprint   

**Figure 8:**
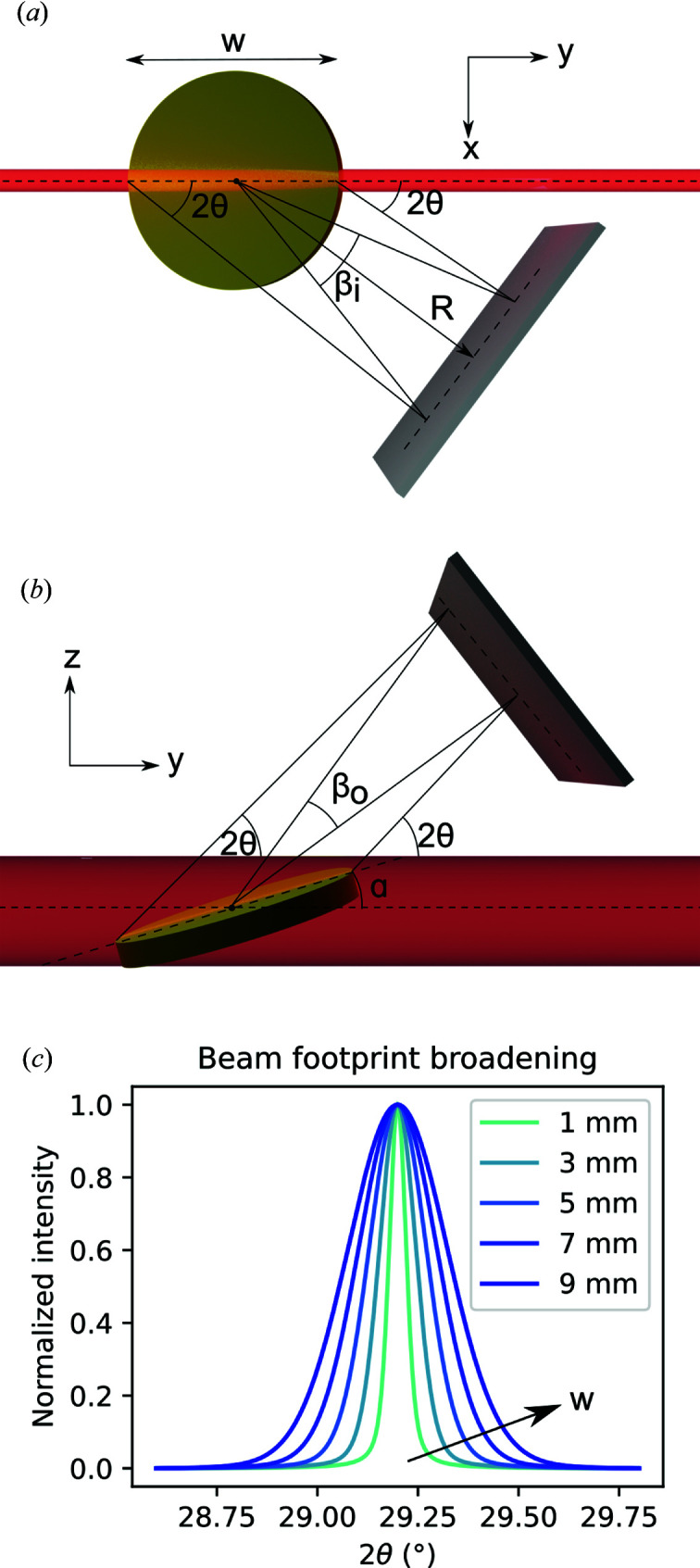
Schematic depiction of the geometric broadening due to the beam footprint at grazing incidence: top view of a γ scan (*a*) and side view of a δ scan (*b*). Simulation of an Au(311) peak at 20 keV, at a grazing angle of 0.1°, scanned out of plane, for increasing sample widths (*c*).

The beam footprint on the sample causes a broadening that affects the instrumental resolution. This is especially true for grazing-incidence geometries, where the beam spills over the sample and illuminates it over its whole length. This geometric effect is depicted in Fig. 8 ▸. Assuming that every volume element illuminated by the beam scatters, all intensity occurring at the diffraction angle 2θ is spread out into a radial range β on the detector. The angular spreads for in-plane scans [Fig. 8 ▸(*a*)] and for out-of-plane scans [Fig. 8 ▸(*b*)] are, respectively, given by 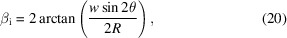


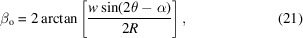
where *w* is the sample width and *R* is the sample-to-detector distance.

Fig. 8 ▸(*c*) shows a simulated intensity profile of an Au(311) peak at 20 keV (2θ = 29.1996°), for different sample widths, assuming an out-of-plane scan and a grazing-incidence angle of 0.1°. The simulated peaks were calculated as Voigt profiles, where the Lorentzian and Gaussian components were determined by the IRF of the I07 beamline at Diamond Light Source (see Section 6.1). The β_o_ broadening was calculated using equation (21) and summed to the Gaussian component of the Voigt profile as follows: 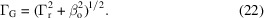



A more insightful way to account for the beam footprint is to determine the peak profile change induced by specimen absorption. Equations (20) and (21) work on the assumption that the intensity of the incoming beam does not change significantly through the whole sample width *w*. This is not always true, since the intensity decays exponentially as described by Beer–Lambert’s law (Swinehart, 1962 ▸). Therefore, a change in diffraction peak profile due to absorption can be modeled as an exponential function. Such effects are well described for several diffraction geometries in the work of Rowles & Buckley (2017 ▸). Once the transmission profile function has been modeled, it can be convoluted with a Voigt or pseudo-Voigt profile in a refining algorithm. This approach not only works for correcting the beam footprint effect but also accounts for possible peak asymmetries.

The beam footprint broadening can be limited by engaging the guard slits between the sample and the detector. If the aperture is small enough, the diffraction originating from a limited region of the sample is detected and the peak broadening is no longer angle dependent but it is rather defined by the slit aperture width. A description of the geometry involving detector slits is available in Appendix *A* [App appa].

### 5.3. Rietveld refinement   

Rietveld refinement (also known as ‘profile refinement’; van Laar & Schenk, 2018 ▸) is a well established analysis method for PXRD data and it is widely employed in the characterization of polycrystalline materials (Rietveld, 1966 ▸, 1967 ▸; Young, 1993 ▸; Loopstra & Rietveld, 1969 ▸; van Laar & Yelon, 1984 ▸). This method consists of fitting the experimental data with a calculated intensity profile which is based on the structural parameters of the material. A nonlinear least-squares algorithm (or other optimization strategy) finds the parameters for a theoretical profile that best matches the experimental intensities. The Rietveld method can be used to find unit-cell parameters, phase quantities, crystallite size and strain, atomic coordinates, and texture. Furthermore, it is possible to model texture and preferred orientations, for example using spherical harmonics (Whitfield, 2009 ▸), although this is outside the scope of the present article.

The quality of the data and having a good starting model are what mainly determine the success of the refinement. For a good PXRD measurement the X-ray beam size should be slightly larger than the crystallite size. In this way, the statistical significance of the detected intensity is maximized because of the larger interaction volume, which leads to the formation of homogeneously continuous diffraction rings. In common PXRD setups the sample is spun to facilitate the measurement of uniform rings, and this is also possible with (2 + 3)-type diffractometers by rotating φ_h_ in the horizontal geometry or φ_v_ in the vertical geometry.

To perform Rietveld analysis, software like *FullProf* (Rodríguez-Carvajal, 1993 ▸), *GSAS-II* (Toby & Von Dreele, 2013 ▸) and *DIFFRAC.SUITE TOPAS* (Coelho *et al.*, 2011 ▸) has been developed. In our somewhat unconventional case, where the data have been manually corrected by Lorentz and polarization factors, *e.g.* by using equation (14), it is possible to prevent *FullProf* from applying further instrumental corrections by selecting ‘Lorentz Polarization not performed’ as the diffraction geometry. However, we could not determine how to disable the instrumental corrections in *GSAS-II* without modifying the Python code making such corrections. We did not test if this was possible in *TOPAS*. Another option would be to multiply the corrected data by the inverse Lorentz–polarization factors used in software such as *GSAS* and *TOPAS*. In this way, it should be possible to use these programs for Rietveld refinement of data collected on a (2 + 3) diffractometer.

In the absence of preferred orientations, equation (5) describes the proportionality between the intensity of a diffraction pattern and the square modulus of the structure factor. Any deviations from this proportionality can be attributed to preferred orientations and refined in software like *FullProf*. These deviations due to preferred orientations can be modeled by, for example, the modified March function (Dollase, 1986 ▸). More complex models involve the generalized spherical-harmonic description (Sitepu, 2002 ▸). In order to find further evidence of preferred orientations, one could plot the intensity as a function of 2θ and χ, as in Fig. 6 ▸. This complete view of all the data gathered in a detector scan enables the search for qualitative evidence of preferred orientations, such as intensity variations along the Debye–Scherrer rings. From the orientation of the scattering vector, it should be possible to quantify the preferred orientation.

#### 5.3.1. Refinement of a NIST LaB_6_ reference   

The LaB_6_ powder is a standard reference material commonly used in powder diffraction for the calibration of diffraction line positions and shapes. A sample of this powder was encapsulated in a borosilicate capillary of 0.5 mm radius, mounted vertically (*i.e.* with the capillary axis parallel to the *z* axis) and measured in a series of γ radial scans in the range 5–60°. In order to measure continuous powder rings, the capillary was rotated by 0.5° along the *z* axis (*i.e.* increasing φ_h_ in Fig. 2 ▸) between each scan. A total of 135 γ scans were combined, each one at a different φ_h_.

**Figure 9:**
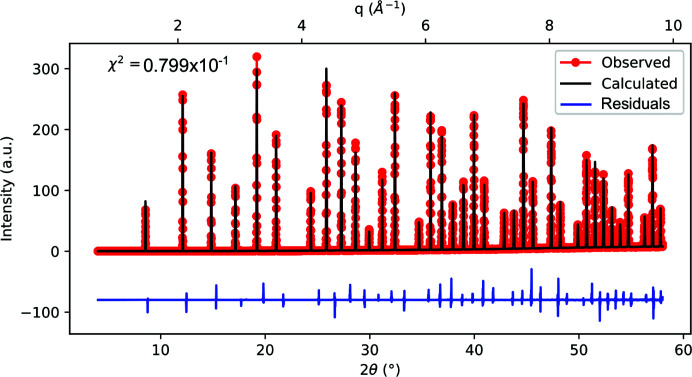
Rietveld refinement of a NIST LaB_6_ SRM 660c PXRD pattern, measured with a 20 keV X-ray beam, performed with *Fullprof* using the pseudo-Voigt line shape. Before the refinement, the data were corrected by Lorentz and polarization factors, flat-detector corrections and interaction volume of X-rays with a capillary. The background was subtracted by fitting a Chebychev polynomial (16 coefficients).

The angle calculations presented in Section 3 were used to plot intensity against 2θ in the one-dimensional pattern shown in Fig. 9 ▸ and to correct the data by the Lorentz–polarization factor, flat-detector effects and interaction volume from a capillary in transmission geometry (see Table 1 ▸). Therefore, the corrected data are proportional to the squared modulus of the structure factors and the multiplicity of each diffraction peak. Since the LaB_6_ standard has very well defined unit-cell parameters, the positions of the diffraction peaks were used to calibrate the sample-to-detector distance and the nominal position of the γ and δ motors.

The good agreement of the calculated model with the observed data is indicated by fairly low residuals (see Fig. 9 ▸). This shows that the setup and data processing employed in this work can be used not only to investigate lattice parameters and phases, but also to record meaningful intensities proportional to structure factors and symmetry multiplicity. Deviations from this proportionality are a clear sign of texture and preferred orientation.

Rietveld refinement of a standard material thin film measured at grazing incidence would be an interesting topic of discussion. However, the fabrication of such thin films often leads to sample morphology with prohibitive roughness. A film of LaB_6_ on a silicon wafer was produced in this work (the fabrication is described in Section 6.2). Owing to the high roughness of this film, the control over the incidence angle, the interaction volume and the refraction effect was limited. The fact that Rietveld refinement was not feasible for this particular sample does not exclude the possibility that it would be possible with a smooth polycrystalline film. In this case, it is recommended to select a grazing-incidence angle α above the critical angle of the substrate, in order to avoid diffraction generated by the reflected beam, which would lead to two overlapping diffraction patterns with a shift of 2α.

## 6. Further examples   

The pattern in Fig. 9 ▸ was used to determine the IRF of the I07 beamline at Diamond Light Source. Furthermore, a different LaB_6_ powder sample (Sigma–Aldrich, grain size 10 µm), prepared by spin coating on an Si substrate, was measured in grazing-incidence and in Bragg–Brentano geometry.

### 6.1. Instrumental resolution function of the I07 beamline   

As discussed in Section 5.1, the peak widths contain instrumental as well as specimen-related information. Although software like *FullProf* and *GSAS-II* has built-in options to refine such parameters, a precise knowledge of the IRF is required to obtain significant information. In this work, we processed the data from the NIST LaB_6_ standard described in the previous section to calculate the IRF.

**Figure 10:**
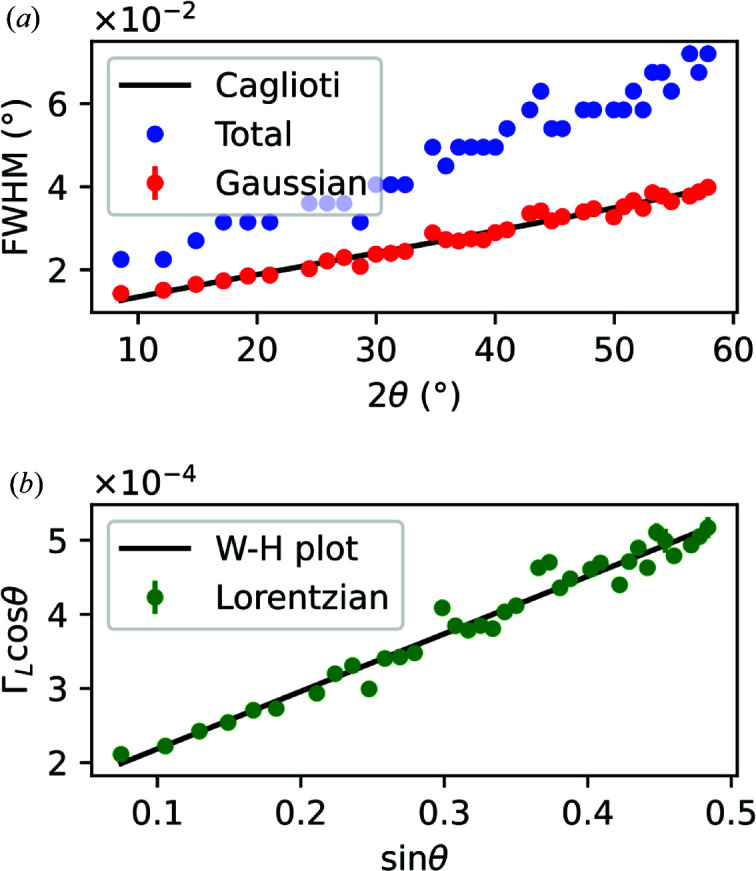
Peak broadening analysis of an LaB_6_ standard reference material. The total FWHMs are plotted together with the Gaussian FWHMs, which are fitted by a Caglioti function [see equation (15)], in (*a*), while the Lorentzian FWHMs, multiplied by 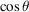, are fitted by a Williamson–Hall plot in (*b*).

All the peaks in Fig. 9 ▸ were fitted one by one with a Voigt profile. The FWHM reported in Fig. 10 ▸(*a*) shows a broadening that has both Gaussian and Lorentzian contributions. The Gaussian and Lorentzian FWHMs of the peak widths were extracted and are reported in Fig. 10 ▸ in the form of a Caglioti function (*a*) and a Williamson–Hall plot (*b*). The dominance of the Gaussian component in the broadening can be explained by the large average crystallite size, *i.e.* above 1 µm as certified by NIST. Because of this large crystallite size, the Scherrer contribution to the Lorentzian broadening is negligible and outside the limits imposed by coherent scattering domains (Miranda & Sasaki, 2018 ▸). Furthermore, since the unit cell of LaB_6_ is not known to show inhomogeneous strain features, we can assume that Γ_s_ in equation (17) is zero and that the Gaussian broadening in Fig. 10 ▸(*a*) is only due to instrumental contributions. Therefore, fitting the Gaussian widths with the Caglioti function will produce a triplet of *U*, *V* and *W* values which describe the IRF. Note that the result of the fitting is affected by the choice of the units expressing the angle. In this work, the Gaussian FWHMs were expressed in degrees. Furthermore, the Williamson–Hall plot in Fig. 10 ▸(*b*), based on equation (19), provides *X* and *Y* values which are characteristics of the X-ray beam bandwidth and the specimen grain size, respectively. All these fitting parameters are reported in Table 2 ▸ with their respective standard errors.

### 6.2. Refraction at grazing incidence   

**Figure 11:**
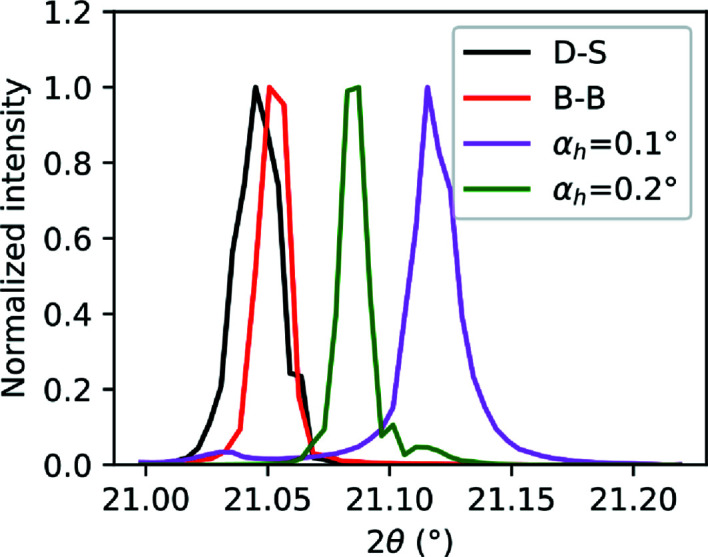
LaB_6_(211) peak for different experimental geometries: Debye–Scherrer (D-S), Bragg–Brentano (B-B), and GIXRD out of plane at α = 0.1 and 0.2°. The shift decreases with increasing incidence angle and can be explained by refraction inside the LaB_6_ film.

A sample of LaB_6_ spin-coated on an Si(100) substrate was measured in Bragg–Brentano and grazing-incidence geometries, using a beam energy of 20 keV. The sample was prepared by mixing 500 mg of an LaB_6_ powder (Sigma–Aldrich, nominal grain size = 10 µm) with 10 mg of ethyl cellulose [Sigma–Aldrich, 48.0–49.5%(*w*/*w*) ethoxyl basis] as a binding agent, in 2 ml of ethanol. The mixture was spin-coated onto a 6 mm square of Si(100) at 800 r min^−1^. Fig. 11 ▸ shows the LaB_6_ (211) peak for the different geometries employed in this work, namely Debye–Scherrer (taken from the capillary data in Section 5.3), Bragg–Brentano, and GIXRD at fixed grazing incidence of 0.1 and 0.2°. The plots exhibit a shift in the peak position from the theoretical diffraction angle, 2θ_calc_, which can be explained by refraction of light in the LaB_6_ film covering the Si substrate. For small incidence angles and a thin film with material constant δ, Lim *et al.* (1987 ▸) modeled this shift as follows: 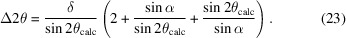



The positions of the peaks in Fig. 11 ▸ for α_h_ = 0.1 and 0.2° were calculated in terms of the center of mass and used to calculate the experimental peak shift by subtracting 2θ_calc_ = 21.0467° for LaB_6_(211). The experimental shift is reported against the theoretical shift calculated using equation (23) in Table 3 ▸. The discrepancy between the two sets of values can be explained by density inhomogeneities in the LaB_6_ film, which also contains ethyl cellulose for the practical purpose of consolidating the film. Furthermore, the LaB_6_ used for this layered sample is not a standard and it could have a slightly different lattice parameter from that reported in the literature after the dissolution in ethanol. Although this kind of sample is not recommended for calibration of XRD setups, it could be used as a reference sample to determine instrumental broadening at grazing incidence. In order to calibrate the detector distance and the motor positions accurately, a well calibrated standard is always recommended.

Equation (23) provides a way of correcting the data which is especially significant in strain analysis. In fact, the homogeneous strain ε is calculated as the relative deviation of the atomic spacing *d* in the strained material from the atomic spacing *d*_0_ in the ideally unstrained material: 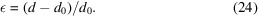
This means that a polycrystalline strained material measured in grazing-incidence geometry will diffract at 2θ angles that slightly deviate from the values predicted by Bragg’s law, not only because of refraction but also because of strain. If the strain of such a material needs to be determined, equation (23) helps to correct for refraction peak shifts.

## 7. Conclusions   

The angle calculations presented in this work describe how to convert data measured by a (2 + 3) diffractometer equipped with an area detector into a one-dimensional XRD pattern, more familiar to the chemistry and materials science communities. We also collected some dispersed knowledge on the phenomena contributing to the peak widths and on the intensity corrections. Since the calculations and the corrections are energy independent, they can be applied to data collected at beamlines operating with hard X-rays (8–30 keV) as well as with high energies (40–150 keV). The Python code used in this work for such corrections and angle calculations is available on Github (https://github.com/giuseppe-abbondanza/pyLjus).

Quantitative PXRD using surface diffractometers is not often done. This work should, however, facilitate quantitative studies using such instruments, including for example *in situ* studies under grazing-incidence conditions.

Furthermore, the calculations presented in this work can be modified to apply to diffractometers with other geometries [*e.g.* (2 + 2), four-circle and six-circle], and they can even apply to setups where the detector is not mounted on a motor and therefore has a fixed position, facing the direct beam. In this configuration, one can assume that effectively γ = δ = 0°.

## Supplementary Material

Rietveld powder data: contains datablock(s) profile1. DOI: 10.1107/S1600576721006245/kc5126sup1.rtv

Rietveld powder data: contains datablock(s) profile1. DOI: 10.1107/S1600576721006245/kc5126sup2.rtv

## Figures and Tables

**Figure 12:**
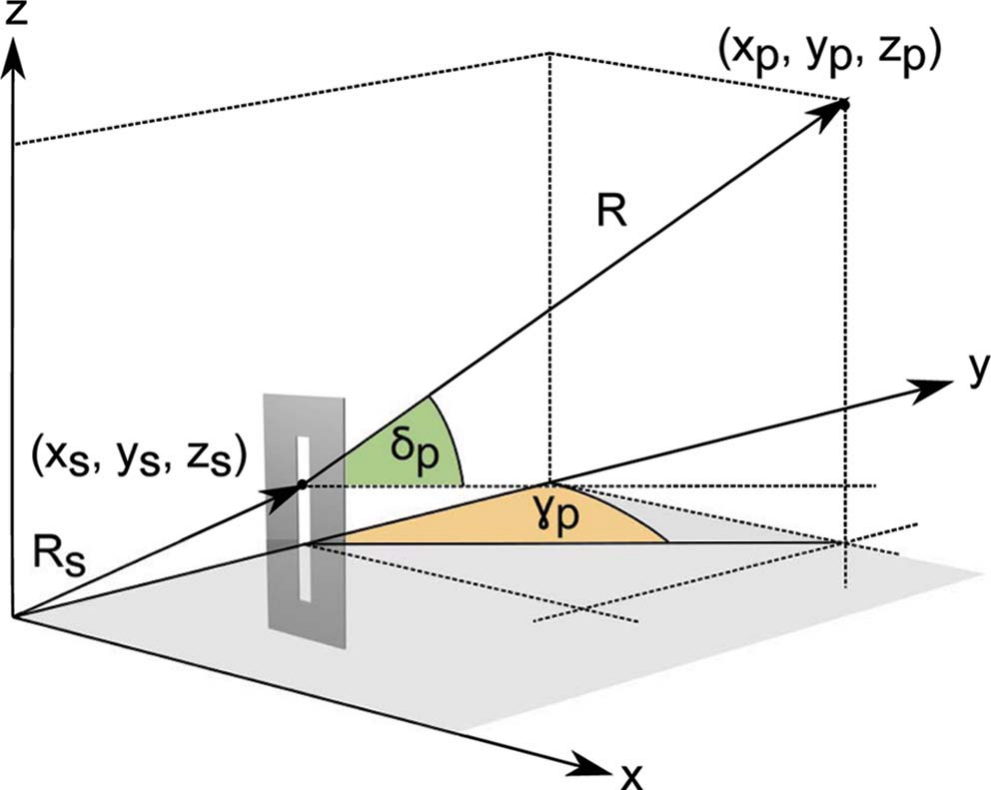
Diagram showing how the detector angles are affected by the use of guard slits.

**Table 1:** Volume corrections for some common experimental geometries

Geometry	Equation	Note	References
Flat-plate, symmetric (Bragg–Brentano)	V_{\rm SR} = {{A} / {(2\mu)}}	–	Cheary *et al.* (2004 ▸)
			Egami & Billinge (2003 ▸)

Flat-plate, asymmetric (grazing incidence)	V_{\rm AR} = ({{A}/{\mu}})\left(1+{{\sin\alpha}/{\sin\gamma}}\right)^{-1}	–	Toraya *et al.* (1993 ▸)
			James (1967 ▸)

Flat-plate with finite thickness	V = 2\left(1+{{\sin\alpha} /{\sin\gamma}}\right)^{-1} \left\{1-\exp\left[-\mu t({{1}/ {\sin\alpha}}+{{1} / {\sin\gamma}})\right]\right\rbrace}	*t* = thickness	Egami & Billinge (2003 ▸)
		Symmetric geometry when α = γ	

Capillary in transmission (Debye–Scherrer)	A(\theta) = A_{L}\cos^{2}(\theta)+A_{B}\sin^{2}(\theta)	*z* = 2μ*r*	Dwiggins (1972 ▸)
	A_{L} = 2{I_{0}(z)-L_{0}(z)-{[{I_{1}(z)-L_{1}(z)}] /{z}}}	*I*_ν_ = νth-order modified Bessel function	Sabine *et al.* (1998 ▸)
	A_B = [I_1(2z) - L_1(2z)]/z	*L*_ν_ = νth-order modified Struve function	

**Table 2:** Fitting parameters of the Caglioti function (*U*, *V*, *W*) and of the Williamson–Hall plot (*X*, *Y*), with their respective standard errors

	Value	Standard error
*U*	2.6912 × 10^−3^	7.1727 × 10^−4^
*V*	1.2460 × 10^−3^	4.8220 × 10^−4^
*W*	5.2366 × 10^−5^	7.3779 × 10^−5^
*X*	7.7621 × 10^−4^	2.4310 × 10^−5^
*Y*	1.4088 × 10^−4^	8.3376 × 10^−6^

**Table 3:** Experimental and theoretical Δ2θ due to refraction in an LaB_6_ film

Incidence angle (°)	α = 0.2	α = 0.1
Experimental shift (°)	0.03841	0.07142
Theoretical shift (°)	0.03488	0.06909
Relative error (%)	9.19	3.27

## Appendix A

Here we present the coordinate transformations to assign a diffraction angle 2θ and an azimuthal angle χ to every pixel of an area detector, given the distance *R* of the detector from the center of the diffractometer and the nominal detector angles γ and δ. The angles and the wavevectors involved in the calculations are schematically shown in Fig. 3 ▸. The following derivation is based on the reciprocal-space coordinate calculations given by Schlepütz *et al.* (2011 ▸).

These calculations are valid for both horizontal geometry and vertical geometry. In both cases, the laboratory frame of reference has its zero coordinates on the diffractometer center and the *y* axis points in the direction of the synchrotron beam. Therefore, the wavevector of the incoming X-ray beam is

where *k* = 2π/λ is the magnitude of the wavevector and λ is the wavelength of the X-ray beam. Similarly, a generic scattered wavevector such as the one in Fig. 3 ▸(*a*) can be represented as

Under the assumption that the scattering is elastic (*i.e.* ∣**k**∣ = ∣**k**′∣ = *k*), the angle 2θ can be found using the definition of the scalar product as follows:

The azimuthal χ angle can be calculated as

Therefore, when δ and γ are known for each pixel of the detector, it is possible to assign a 2θ and a χ value to each pixel. To this end, we need to calculate the exact position of the detector center.

We define the detector center as the point on which the direct beam impinges when the detector is in the zero position (*i.e.* when γ = δ = ν = 0, as shown in Fig. 2 ▸). In this position, the coordinates of the detector center are given by calculating the ‘center of mass’ of an image recorded while the direct beam impinges on the detector:

where (*i*, *j*) are the detector pixel coordinates, **c** is the vector pointing to the center of mass, *S* is the sum of all intensities detected in the image, and *I*(*i*, *j*) and  are the intensity and the vector describing the position of the (*i*, *j*) pixel.

Let us denote the coordinates of the detector center found in equation (31) by (*c*_*x*_, *c*_*z*_). A pixel with coordinates (*i*, *j*) has an offset (Δ*x*, Δ*z*) from the detector center given by

where *w*_*x*_ and *w*_*z*_ are the width of the pixel along the **x** and **z** directions, respectively (for a Pilatus 100K detector *w*_*x*_ = *w*_*z*_ = 172 µm). Fig. 3 ▸(*b*) illustrates the typical offsets in pixel position described above. Here we assign the (0, 0) coordinates to the upper-left detector pixel. When the detector is at the zero position, the position of a generic (*i*, *j*) pixel in the laboratory coordinates is simply

For nonzero detector angles, the new (*x*_p_, *y*_p_, *z*_p_) coordinates of the (*i*, *j*) pixel are found by rotating the vector (Δ*x*, *R*, Δ*z*) by γ, ν and δ around the *z*, *y* and *x* axes, respectively. Therefore, the pixel position in the laboratory frame of reference becomes

where **Γ**, **Δ** and **N** are the matrices describing the rotations around the *z*, *x* and *y* axes, defined as follows:

The order of matrix multiplication depends on how the diffractometer is built up. Since the γ circle holds the δ circle, which in turn sustains the ν motor, the order of matrix multiplication is **R**_*z*_, **R**_*x*_ and **R**_*y*_.

The angle values for a generic pixel γ_p_ and δ_p_ can be calculated as follows:

where *d* is the distance of the pixel from the center of the diffractometer, given by

### A1. Detector slits

Apertures and slits are used in PXRD to control the beam size, divergence and angular resolution, as well as to isolate the diffracted signal of the sample from the background caused by the sample environment. High-density metals are often used in the fabrication of slits owing to their low X-ray transmission. When the detector slits are engaged, part of the diffracted X-ray beam is blocked and the effect of the beam footprint on the peak broadening is reduced (see Section 5.2). The detected signal can be seen as originating from a point within the slit aperture rather than from the diffractometer center. Therefore, we can derive a new set of ‘effective’ detector angles (γ_p_, δ_p_), as illustrated in Fig. 12 ▸.

The ν rotation axis is always perpendicular to the aperture plane for every nominal detector angle (γ, δ), *i.e.* the slit aperture rotates with the detector. Under this assumption, the coordinates of the aperture in the laboratory coordinate system are given by

where *R*_s_ is the slit distance from the diffractometer center. The effective detector angles are now dependent on the distance between the pixel and the slit and are given by

### A2. Vertical geometry

In the vertical geometry the surface normal of the sample and the *y* axis lie in the horizontal plane, while the *x* axis points upwards. The equations for the incoming beam wavevector and those for the offset from the detector center are the same as in the horizontal geometry, *i.e.* equations (25), (32) and (33), while the generic scattering vector is given by

Since the *y* component has not changed, the equation for the 2θ angle is the same as equation (29). The equation for the χ angle (30) is also the same in both geometries. The rotation matrices, however, are different in this frame of reference and are given by

As in the case of horizontal geometry, equation (35) applies to the calculation of pixel coordinates.
